# Using autoregressive integrated moving average (ARIMA) models to predict and monitor the number of beds occupied during a SARS outbreak in a tertiary hospital in Singapore

**DOI:** 10.1186/1472-6963-5-36

**Published:** 2005-05-11

**Authors:** Arul Earnest, Mark I Chen, Donald Ng, Leo Yee Sin

**Affiliations:** 1 Department of Clinical Epidemiology, Tan Tock Seng Hospital, Singapore; 2 Communicable Disease Centre, Singapore

## Abstract

**Background:**

The main objective of this study is to apply autoregressive integrated moving average (ARIMA) models to make real-time predictions on the number of beds occupied in Tan Tock Seng Hospital, during the recent SARS outbreak.

**Methods:**

This is a retrospective study design. Hospital admission and occupancy data for isolation beds was collected from Tan Tock Seng hospital for the period 14^th ^March 2003 to 31^st ^May 2003. The main outcome measure was daily number of isolation beds occupied by SARS patients. Among the covariates considered were daily number of people screened, daily number of people admitted (including observation, suspect and probable cases) and days from the most recent significant event discovery. We utilized the following strategy for the analysis. Firstly, we split the outbreak data into two. Data from 14^th ^March to 21^st ^April 2003 was used for model development. We used structural ARIMA models in an attempt to model the number of beds occupied. Estimation is via the maximum likelihood method using the Kalman filter. For the ARIMA model parameters, we considered the simplest parsimonious lowest order model.

**Results:**

We found that the ARIMA (1,0,3) model was able to describe and predict the number of beds occupied during the SARS outbreak well. The mean absolute percentage error (MAPE) for the training set and validation set were 5.7% and 8.6% respectively, which we found was reasonable for use in the hospital setting. Furthermore, the model also provided three-day forecasts of the number of beds required. Total number of admissions and probable cases admitted on the previous day were also found to be independent prognostic factors of bed occupancy.

**Conclusion:**

ARIMA models provide useful tools for administrators and clinicians in planning for real-time bed capacity during an outbreak of an infectious disease such as SARS. The model could well be used in planning for bed-capacity during outbreaks of other infectious diseases as well.

## Background

Early isolation of infectious cases has been shown to be a key component for the successful management of SARS outbreaks[1]. Due to the potential for nosocomial transmission[2-6], the imperfect ability of clinical criteria to distinguish cases of SARS at presentation[7], the possibility of atypical presentations[8,9], and the lack of a sensitive diagnostic test in early disease[10], front-line clinicians need to err on the side of caution when admitting cases, and isolate patients until SARS can be clinically and virologically ruled out. As a result, the number of admissions and isolation beds required during management of SARS outbreaks can be expected to significantly exceed that used for actual SARS cases.

While hospitals are generally built with a fixed ratio of isolation to general ward beds, surge capacity for isolation beds can be met by conversion of single room wards, decanting of existing patients with lesser indications for isolation, and activation of isolation facilities at alternative sites and institutions. However, these processes require time, and the ability to forecast requirements is hence a critical component of efficient outbreak management.

During SARS outbreak in Singapore from 1 Mar to 31 May 2003, the Communicable Disease Centre (CDC) was the initial designated facility for the screening management of all SARS cases, beginning on 14 Mar 2003, two days after the WHO alert was sounded on 12 Mar[11]. To accommodate the surge in cases, the parent hospital of the CDC, Tan Tock Seng Hospital (TTSH) became designated as the central facility for management of all SARS cases in Singapore from 22 Mar 2003[12]. As a result of the above policy, 231 of 238 SARS cases diagnosed during the Singapore outbreak were admitted to TTSH. TTSH bed utilization patterns here hence reflect national level requirements for outbreak management. Various papers have described qualitative aspects of hospital management during SARS[13-15], but none have provided quantitative tools for predicting requirements for isolation beds. Yet such quantitative models would be of great utility to both hospital administrators and national level planners during outbreak management.

The main objective of this study is to apply autoregressive integrated moving average (ARIMA) models to make real-time predictions on the number of beds occupied in TTSH during the SARS outbreak, starting from 14 Mar 2003, when the CDC was activated, to 31 May 2003 when Singapore was declared SARS free.

## Methods

This was a retrospective study design. Hospital admission and occupancy data for isolation beds was collected from Tan Tock Seng hospital for the period 14^th ^March 2003 to 31^st ^May 2003. The main outcome measure was daily number of isolation beds occupied by SARS patients, including those fulfilling WHO criteria for suspect and probable SARS[16], as well as those admitted not fulfilling WHO case definitions but admitted to isolation rooms for observation. Among the covariates considered were daily number of people screened, daily number of people admitted (including observation, suspect and probable cases) and days from the most recent significant event discovery. Key events considered were as follows:

1. 14^th ^Mar: discovery of the TTSH outbreak

2. 22^nd ^Mar: press release that TTSH would dedicated to SARS management

3. 4^th ^Apr: discovery of an outbreak at Singapore General Hospital (SGH)

4. 11^th ^Apr: discovery of an outbreak at National University Hospital (NUH)

5. 20^th ^Apr: discovery and press release on an outbreak at Pasir Panjang Wholesale Market (PPWM)

6. 13^th ^May: discovery of a cluster of febrile staff and patients at the Institute of Mental Health (IMH)

Details on the above can be found in the chronology of press releases on SARS events in Singapore[17]. Events 1–5 all involved probable SARS cases, whereas event 6 proved to be a false alarm[18].

We utilized the following strategy for the analysis. Firstly, we split the outbreak data into two. Data from 14^th ^March to 21^st ^April 2003 was used for model development. We used structural ARIMA models in an attempt to model the number of beds occupied[19]. Estimation is via the maximum likelihood method using the Kalman filter[20]. For the ARIMA model parameters, we considered the simplest parsimonious lowest order model.

We computed various permutations of the order of correlation (AR), order of integration (I) and order of moving average (MA), and chose the optimal combination of parameters using the mean square error. The correlogram and partial correlogram graphs were also used to help in deciding the order of moving average (MA) and auto-regressive (AR) terms to include in the model. To ensure the model was robust to symmetric nonnormality in the disturbances, including heteroskedasticity, we computed Huber/White/sandwich estimator of variance for the coefficient estimates[21]. Before modeling the bed occupancy, we examined whether the series was stationary. In the event of non-stationarity, we opted to set an a-prior value of 1 for starting the Kalman recursions[22].

We used the likelihood ratio test to determine if inclusion of other covariates helped improve the fit of the model. Based on the final model selected, we assessed the out-sample validity of the model, by applying the model to predict the number of beds occupied for the remaining period of the outbreak (i.e. 22^nd ^April 2003 to 31^st ^May 2003). In addition, we also made three-day forecasts for selected periods during the outbreak, starting from day 4 of the outbreak. We used the mean absolute percentage error (MAPE) to measure and quantify the quality of fit. A lower MAPE value will indicate a better fit of the data. All tests were conducted at the 5% level of significance, and data analysis was performed in Stata V7.0 (Stata Corporation, College Station, TX, USA).

## Results

**Figure 1:**
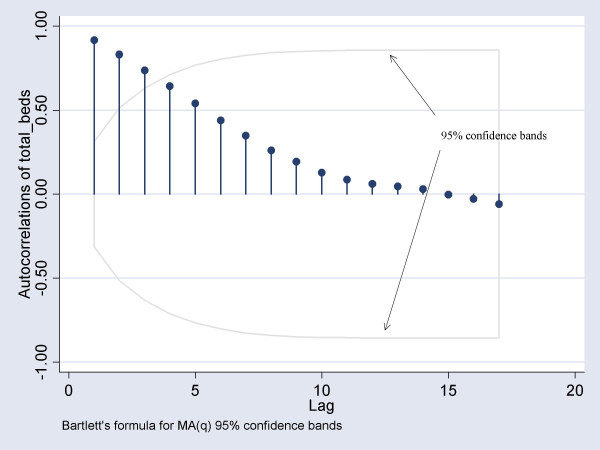
Correlogram of total beds occupied

**Figure 2:**
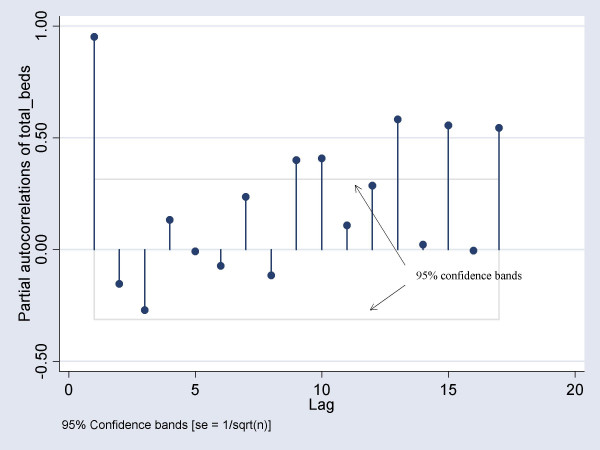
Partial correlogram of total beds occupied

From 14^th ^March 2003 to 31^st ^May 2003, the median daily number of beds occupied was 134 (IQR: 105–193). The range was 15 to 238 beds. The number of beds occupied reached it's peak on the 24^th ^and 28^th ^of April 2003, with a total of 238 beds. For the final ARIMA model, we found that the ARIMA (1,0,3) model was the most suitable, with an auto-regression term of 1 and a moving average term of 3. The correlogram indicated that there was a significant autocorrelation out to about 3 lags, and this autocorrelation decayed slowly over time (figure 1). The partial auto correlation function (PACF) plot suggested that the only highly significant partial autocorrelation occurred at one lag (figure 2). We found that the AR(1) coefficient of 1.02 and MA(3) coefficient of -0.95 were significant (p < 0.05). The likelihood ratio test indicated that the total number of admissions on the previous day and number of probable cases admitted on the previous day were significant predictors, and these variables were thus included in the final model. Furthermore, the estimated variance of the white-noise disturbance was found to be 4.47 (see table 1). Days from most recent significant event discovery and number of patients screened were not found to be significant predictors of daily number of isolation beds occupied.

**Table 1:** Parameters for the final ARIMA model

**Variable**	**Coefficient**	**95 % CI**	**p-value**
Constant	17.03	6.43, 27.62	0.002
Previous day's total admissions	-0.37	-0.63, -0.11	0.006
Previous day's probable case admissions	1.17	0.37, 1.97	0.004
			
ARMA Parameters			
AR (1)	1.02	0.98, 1.06	<0.001
MA (1)	-1.34	-2.94, 0.27	0.102
MA (2)	-0.96	-1.80, -0.12	0.026
MA (3)	-0.95	-1.52, -0.38	0.001
Sigma	4.47	1.40, 7.53	0.004

**Figure 3:**
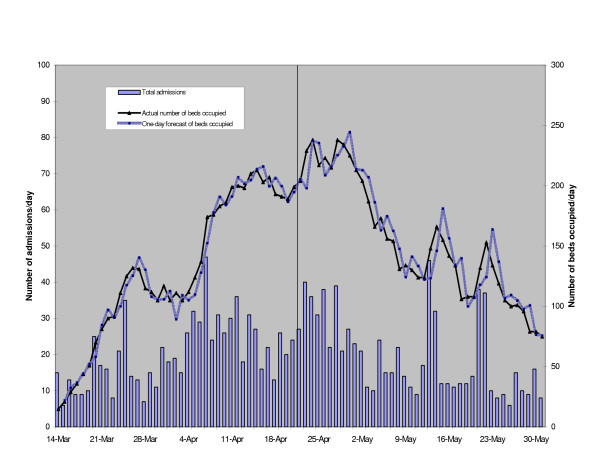
Admissions, predicted and actual number of beds occupied

As we can see from figure 3, the predictions from the ARIMA model performed reasonably well, both for the training and validation data. The MAPE for the training set and validation set were 5.7% and 8.6% respectively. This translated to an error rate of ± 7 beds and ± 13 beds respectively. We have also provided the model parameters and their corresponding MAPE values for some of the alternative ARIMA models that we had considered (table 2).

**Table 2:** Comparison of various selected ARIMA models

**Model**	**Training Set MAPE**	**Validation Set MAPE**
ARIMA (1,0,0)	6.0%	9.0%
ARIMA (1,0,1)	6.2%	9.2%
ARIMA (1,0,2)	5.9%	8.4%
**ARIMA (1,0,3)**	**5.7%**	**8.6%**
ARIMA (1,0,4)	5.3%	13.1%
ARIMA (1,1,3)	5.3%	18.7%
ARIMA (0,0,2)	9.8%	32.8%
ARIMA (0,0,3)	9.5%	16.4%

For three-day predictions, we found that the model fared reasonably well (see table 3). For day 4 to day 6 of the outbreak, the error rate was 6%. For day 7 to day 9, the rate was 10%, day 10 to day 12, 7% and finally, for day 13 to day 15, it was 9%. Although the MAPE values were within reasonable levels, we note that generally, the model under-predicts in the early stage of the outbreak, and over-predicts in the later stage of the outbreak (tables 3 and 4).

**Table 3:** Forecast of bed occupancy in the initial stage of the outbreak

**Day of outbreak**		**3rd Day**	**6th day**	**9th Day**	**12th Day**	**MAPE**
**Three-day forecast**	**Actual number of beds**					
Day 4	36	37				6
Day 5	44	42				
Day 6	51	46				
						
Day 7	70		60			10
Day 8	81		72			
Day 9	90		87			
						
Day 10	92			99		7
Day 11	111			108		
Day 12	125			111		
						
Day 13	132				116	9
Day 14	131				122	
Day 15	115				124	

**Table 4:** Forecast of bed occupancy in the late stage of the outbreak

**Day of outbreak**		**67th Day**	**70th day**	**73rd Day**	**76th Day**	**MAPE**
**Three-day forecast**	**Actual number of beds**					
Day 68	108	97				22
Day 69	132	106				
Day 70	153	96				
						
Day 71	134		129			16
Day 72	119		134			
Day 73	105		136			
						
Day 74	100			112		17
Day 75	101			119		
Day 76	96			117		
						
Day 77	79				103	35
Day 78	79				106	
Day 79	75				105	

## Discussion

To the best of our knowledge, this is the first study to suggest the application of a known statistical method such as the ARIMA model, to predict and monitor the utilization of hospital isolation beds during the recent SARS outbreak in Singapore, for which Tan Tock Seng Hospital was the designated hospital for all patients presenting with SARS-like symptoms and exposures.

ARIMA models have traditionally found application in the financial sector. There has been limited literature on their use in healthcare; recent examples include their use in assessment of seasonal variation in selected medical conditions[23], and as a surveillance tool for outbreak detection[24]. There has been some research indicating that time series modeling may be more appropriate than the simple trend fitting approach, which suffers from model specification error[25]. ARIMA models have been used to forecast attendance at accident and emergency departments in the United Kingdom. Particularly, researchers have shown that the forecasting methodology can be improved by incorporating the ARIMA method[26]. Here, we show that the ARIMA model can be used over the much shorter time-frame of a single outbreak to forecast bed-utilization. The three-day forecasts from the model are fairly reasonable. The MAPE is low, allowing planners to confidently decide, with sufficient lead-time, on the need to open new isolation wards, each of which, in our setting, holds between 10 to 20 patients.

However, the model has its limitations. Firstly, unmeasured confounders could have affected the results of this study, although we have accounted for measured confounders by incorporating significant covariates into the final model. Secondly, the most significant covariate was the number of probable SARS cases admitted. This is not surprising, as probable SARS cases stayed longer in isolation facilities compared to cases which turned out not to be SARS (unpublished data). In this analysis, we used the final classification for each case after a variable period of observation and investigation, and not the admission classification.

This approach was chosen, as time required to confirm cases will be likely be shortened in any future outbreaks, in view of various advancements for SARS diagnostics[27,28]. However, it is still uncertain what proportion of SARS cases can be accurately classified on admission, and this may affect model performance. Another point to note is that the model generally under-predicts the number of beds occupied in the early stage of the outbreak and over-predicts at the later stage of the outbreak for three-day forecasts. One possible explanation could be that the model was derived from the first half of the data and applied to both the first and second halves of the outbreak. It is important for administrators to take this into account during bed planning, perhaps by allowing for appropriate buffer beds. Lastly, the ARIMA model parameters may differ under different practice protocols within different outbreak settings as well as between different SARS afflicted countries. It would be useful to calibrate the model using individual country level data. We would recommend that, in an actual outbreak, real-time calibration be performed, with additional data available on each day fed-back into the model to improve its predictive ability.

The application of ARIMA models in bed utilization is not only useful for an outbreak of SARS and emerging infectious diseases, but also for projecting resource requirements in bioterrorism events. It has been recognized by others that resource requirements will not include just isolation beds, but also outpatient resources[29], pharmaceuticals[30], as well as intensive care facilities[31]; few articles, however, have proposed any models for forecasting such requirements. The challenge, therefore, lies in the collection of timely surveillance and resource utilization data for this specific purpose, peace-time exploration of the most appropriate methods of analysis, and real-time validation and application in the event of an outbreak.

## Conclusion

The ARIMA model that we developed for modeling the number of beds occupied during the SARS outbreak performed reasonably well, with a MAPE of 5.7% for the training set, and 8.6% for the validation set. In addition, we found that three-day forecasts provided a reasonable prediction of the number of beds required during the outbreak

ARIMA models provide useful tools for administrators and clinicians in planning the use of isolation beds during an outbreak of an infectious disease such as SARS. The model could be used in planning for bed-capacity during outbreaks of other infectious diseases, as well as predicting requirements for other critical resources.

## Competing interests

The author(s) declare that they have no competing interests.

## Authors' contributions

DN conceived the study and contributed to the study design, analysis and interpretation. AE contributed to the statistical analysis, interpretation and writing of the manuscript. MIC contributed to the statistical analysis, interpretation and writing of the manuscript. LYS contributed to the interpretation and writing of the manuscript.

## Pre-publication history

The pre-publication history for this paper can be accessed here:


